# Prevalence and associated factors of mental distress among private college nursing students in Gondar town, Northwest Ethiopia, 2023

**DOI:** 10.3389/fpsyt.2025.1544252

**Published:** 2025-09-29

**Authors:** Agegnehu Amare, Niguse Yigzaw, Bizuneh Tesfaye, Enguday Tirfeneh, Seblewongel Tinsae, Kiber Temesgen, Gebremeskel Mesafint

**Affiliations:** 1 Department of Psychiatry, Mizan-Tepi University, Mizan-Aman, Ethiopia; 2 Department of Psychiatry, College of Medicine and Health Sciences, University of Gondar, Gondar, Ethiopia

**Keywords:** mental distress, private college, nursing students, Gondar, Ethiopia

## Abstract

**Background:**

Mental distress (MD) refers to a range of troubling, confusing, or unusual internal experiences and symptoms that affect more than 25% of people during their lives. Students with mental distress are more likely to face negative outcomes, including impaired cognitive functioning, learning difficulties, poor academic performance, and substance use. This study aimed to address a research gap by estimating the prevalence of mental distress and identifying associated factors.

**Methods:**

An institution-based cross-sectional study was conducted in Gondar town using a multistage stratified sampling technique. The Kessler 10-item scale was applied to assess the prevalence of mental distress. Bi-variable and multivariable logistic regression analyses were performed, and variables with a p-value <0.05 in the multivariable model at a 95% confidence interval (CI) were considered statistically significant.

**Results:**

The prevalence of mental distress among private college nursing students was 52.7% (95% CI: 48.8%–56.7%). In the multivariable analysis, factors significantly associated with mental distress were female sex (AOR = 2.8, 95% CI: 1.50–3.16), rural residence (AOR = 1.96, 95% CI: 1.30–2.95), poor social support (AOR = 2.03, 95% CI: 1.20–3.42), not participating in regular physical exercise (AOR = 3.28, 95% CI: 1.68–6.37), never attending guidance and counseling services (AOR = 2.37, 95% CI: 1.12–4.99), and a family history of mental illness (AOR = 2.65, 95% CI: 1.31–5.35).

**Conclusion and recommendations:**

The prevalence of mental distress was notably high. Associated factors included sex, residence, social support, physical exercise, access to guidance and counseling services, and family history of mental illness. We recommend that the Ministry of Health and Ministry of Education strengthen guidance and counseling services and promote regular physical exercise at the college level.

## Introduction

“Mental distress is a range of symptoms and experiences of a person’s internal life that are held to be troubling, confusing, or out of the ordinary” (1) mainly characterized by symptoms such as depression, anxiety, fatigue, insomnia, irritability, forgetfulness, difficulty concentrating, and somatic complaints including sleep problems, headache, and backache (2).

Mental distress is widespread globally; more than 25% of all individuals experience it during their lives (3). Although it affects individuals of all nations regardless of age, sex, or income, college students are particularly prone to negative mental health outcomes (4).

Previous studies have reported a higher prevalence of mental distress among university students in low- and middle-income countries (LMICs) in Asia and sub-Saharan Africa, including Jizan (5), India (6) and Nigeria (7, 8). However, recent studies have also shown a sharp increase in prevalence in high-income countries (9–12).

In Ethiopia, two systematic reviews estimated a pooled prevalence of mental distress indicating that more than one-third of the students had mental distress (13, 14). Similarly, individual studies conducted in higher academic institutions reported prevalence ranging from 19.3% (15) and 53.2% (16–20).

Specifically, at the college level cross-sectional studies conducted in Southern Ethiopia (Hawassa) and Dessie showed that its prevalence was 22.30% and 16.2%, respectively (21, 22).

Although college can be an exciting time for most youth, previous research has demonstrated that the transition from high school to college can be stressful (3). Additionally, private college students often bear full responsibility for managing non-academic issues such as food, housing, transportation, stationery, tuition, and related costs. Many also rent homes and live in groups, which may further contribute to the development of mental distress.

Students with mental distress are more likely to face negative consequences such as impaired cognitive functioning, learning abilities, academic performance (23–25), emotional and interactional abilities (26–30) and are prone to substance abuse (9, 31). They are also at higher risk of depression and anxiety disorders (27), which may impair their functionality later in life (31–33). Early diagnosis and management of mental distress play a crucial role in achieving favorable outcomes (34).

Even though numerous studies have been conducted in Ethiopia to investigate the prevalence and associated factors of mental distress in higher academic institutions, there remains a significant information gap regarding its magnitude and determinants among private college nursing students. Therefore, this study aims to address this gap. Finally, the results of this study will also support educational and health bureaus in implementing early interventions.

## Materials and methods

### Study design, setting, and period

An institutional-based cross-sectional study was conducted from May/23/2023 to June/03/2023 among private college nursing students in Gondar, a town in Northwest Ethiopia. Gondar is the capital of the Central Gondar Administrative Zone and one of Ethiopia’s historic towns, located 657 km northwest of Addis Ababa. The town is home to nine private colleges with a total of 1,872 nursing students in the 2023 academic year.

### Study populations

All undergraduate nursing students enrolled in private colleges in Gondar town constituted the study population.

### Sample size and sampling techniques

The sample size was calculated using a single population proportion formula, with the following assumptions: a 50% expected proportion, 95% confidence interval (CI), 5% margin of error, 10% non-response rate, and design effect of 1.5. Accordingly, the final sample size was determined to be 636.

A stratified multistage sampling technique was used to select a representative sample of the study population. Initially, three colleges were randomly selected from the nine available using simple random sampling. The sample size of 636 was then proportionally allocated to each college. Finally, a computer-generated random sampling technique was applied to select the study subjects from each of the three colleges.

### Data collection procedure and tools

Data were collected from the three randomly selected colleges using a structured, self-administered questionnaire consisting of five parts. The first part assessed socio-demographic and academic characteristics. The second part included the Kessler Psychological Distress Scale (K-10), which was used to measure the prevalence of mental distress. The K-10, developed based on item response theory models (35), consists of 10 questions assessing the frequency of non-specific mental distress during the past month (36). Each question has five possible responses, ranging from “none of the time” (score = 1) to “all of the time” (score = 5).

All responses to the K-10 were summed to obtain a total score. A score of <20 was considered normal, while a score ≥20 indicated the presence of mental distress (37). The tool has been validated by the World Health Organization (WHO) for use in developing countries (38) and has been validated for Ethiopian adults, demonstrating good reliability (39). Its sensitivity and specificity are 84.2% and 77.8%, respectively (40).

The third part of the questionnaire assessed behavioral factors, including substance use (current and lifetime use), which was adapted from the Alcohol, Smoking, and Substance Involvement Screening Test (ASSIST), a well-validated instrument developed by the WHO (41). The history of physical exercise was assessed using one question adapted from the Global School-based Health Survey (GSHS) questionnaire, developed by the WHO and the Centers for Disease Control (41) and Prevention (CDC).

The fourth part of the questionnaire addressed psychosocial factors. These included the level of social support, measured by the Oslo-3 scale, which has a total score range of 3–14 (42). This section also covered the availability and use of guidance and counseling services in the colleges, assessed with simple yes/no questions.

The final part of the questionnaire focused on clinical factors, including chronic medical illness and family (blood-related) history of mental illness, assessed with simple yes or no questions.

### Data quality control

The questionnaire, originally prepared in English, was first translated into Amharic and then back-translated into English by independent individuals with the assistance of psychiatry professionals and returned-back to Amharic for the participants. The pre-test was done on 5% of the total sample size drawn from bazmarry college (one of the non-selected colleges) and the internal consistency of kessler-10 was checked by using Cronbach’s alpha reliability test with a score of 0.88.

Data were collected by three BSc-level psychiatric professionals and supervised by two additional BSc psychiatry professionals after two days of training on study instrument administration. During data collection, supervisors oversaw each site, and meetings were held among the data collectors, supervisors, and the principal investigator to review daily activities. Collected data were checked for completeness, and incomplete responses were discarded.

### Data processing and analysis

After checking, coding, and entry into EpiData version 4.6.0.6, the data were exported to STATA version 14 for analysis. Socio-demographic characteristics and other factors were analyzed using descriptive statistics (percentages, means, and standard deviations).

Bivariable logistic regression analysis was first performed to identify associations between each independent variable and the outcome variable. Variables with a *p*-value <0.20 in the bivariable analysis were entered into the multivariable logistic regression model. A *p*-value of < 0.05 was considered statistically significant and the adjusted odds ratio (AOR) with 95% confidence interval (CI) was calculated. Model fitness was evaluated using the Hosmer–Lemeshow goodness-of-fit test (p = 0.96) and there was no problem of multi-collinearity since every variable had a variance inflation factor (VIF) of less than five and a mean of 1.35.

## Results

### Socio-demographic characteristics of private college nursing students

Of the 636 nursing students approached, 620 provided informed consent and completed the survey, yielding a response rate of 97.5%. Nearly half of the participants, 302 (48.7%), were male. The mean (± SD) age of respondents was 21.86 ± 2.20 years. A majority, 521 (84.0%), identified as followers of Orthodox Christianity, and most, 575 (92.7%), reported participating in religious practices. More than half, 397 (64.0%), came from urban backgrounds, while 258 (41.6%) lived with both parents. Regarding marital status, 541 (87.3%) were single, and a significant proportion, 423 (68.2%), reported experiencing financial difficulties (**Table 1**).

**Table 1:** Socio-demographic characteristics of private college nursing students in Gondar, Northwest Ethiopia, 2023 (n = 620).

Variables	Category	Frequency	Percentage
Age	<=21	305	49.19
	>21	315	50.81
Sex	Male	302	48.71
	Female	318	51.29
Religion	Orthodox	521	84.03
	Muslim	63	10.16
	Others*	26	5.81
Spiritual practice	Yes	575	92.74
	No	45	7.26
Residency	Urban	397	64.03
	Rural	223	35.97
Living with	Both parent	258	41.61
	Single parent	123	19.84
	Lonely	146	23.55
	With spouse	51	8.23
	With friends	37	5.97
	Others**	5	0.81
Marital status	Single	541	87.26
	Married	52	8.39
	Others***	27	4.36
Financial problem	Yes	423	68.23
	No	197	31.77

*Protestant, Catholic, Adventist, and Jewish.

**With relatives and with employers as a house-maids.

***divorced and separated.

### Academic characteristics of private college nursing students

With respect to academic performance, about two-thirds (66.6%) of participants had a cumulative grade point average (CGPA) of ≥3.00. The distribution of students by year of study was as follows: first year, 23.9%; second year, 30.0%; third year, 32.9%; and fourth year, 13.2%. The majority, 484 (78.1%), reported having good job prospects (**Table 2**).

**Table 2:** Academic characteristics of private college nursing students in Gondar, Northwest Ethiopia, 2023 (n = 620).

Variables	Category	Frequency	Percentage
Year of study	1^st^ year	148	23.87
	2^nd^ year	186	30.00
	3^rd^ year	204	32.90
	4^th^ year	82	13.23
CGPA	Satisfactory (<2.5)	50	8.06
	Distinction (>=2.5)	157	25.32
	Great distinction (>=3.00)	212	34.19
	Very great distinction (>=3.5)	201	32.42
Good prospect to find a job	Yes	484	78.06
	No	136	21.94

### Behavioral characteristics of participants

Out of the total study participants, more than two-thirds, 427 (68.9%), reported drinking alcohol at least once in their lives, while 374 (60.3%) reported drinking within the past 3 months. Other forms of substance use were reported by fewer than 5% of participants. Regarding physical exercise, only 59 (9.5%) students reported engaging in regular activity (**Table 3**).

**Table 3:** Behavioral characteristics of participants in Gondar, Ethiopia, 2023 (n=620 respondents).

Variables		Category	Frequency	Percentage
Alcohol	Ever use	Yes	427	68.87
		No	193	31.13
	Current use	Yes	374	60.32
		No	246	39.68
Cigarette	Ever use	Yes	11	1.77
		No	609	98.23
	Current use	Yes	9	1.45
		No	611	98.55
Chat	Ever use	Yes	21	3.39
		No	599	96,61
	Current use	Yes	16	2.58
		No	604	97.42
Others*	Ever use	Yes	6	0.97
		No	614	99.03
	Current use	Yes	4	0.65
		No	616	99.35
Regular physical exercise		Yes	59	9.52
		No	561	90.48

*Marijuana and opioids.

### Psychosocial and clinical characteristics of participants

Fewer than one-quarter, 115 (18.6%), had ever used guidance and counseling services in their colleges, and 448 (72.3%) reported that the service was not accessible. Only 38 (6.1%) participants reported a history of chronic illness, and 64 (10.3%) reported a family history of mental illness. Regarding social support, nearly half (48.4%) of the students had moderate support (**Table 4**).

**Table 4:** Psychosocial and clinical characteristics of participants in Gondar, Ethiopia, 2023 (n=620 respondents).

Variables	Category	Frequency	Percentage
Social support	Poor	201	32.42
	Moderate	300	48.39
	Strong	119	19.19
Attending guidance and counseling	Yes	115	18.55
	No	57	9.19
	Not available	448	72.26
Family history of mental illness	Yes	64	10.32
	No	556	89.68
Chronic illness	Yes	38	6.13
	No	582	93.87

### Prevalence of mental distress

The prevalence of mental distress among private college nursing students was 52.7% (95% CI: 48.8%–56.7%). The prevalence among males was 44.4%, compared with 60.7% among females. Thus, a relatively higher prevalence of mental distress was observed in female students.

### Factors associated with mental distress

Variables including sex, living condition, religion, religious practices, residency, social support, job prospects, CGPA, chronic illness, family history of mental illness, regular physical exercise, guidance and counseling service use, alcohol ever use, and current alcohol use were associated with mental distress at p <0.20 in the bivariable analysis and were entered into the multivariable regression model.

In the multivariable analysis, the following factors were significantly associated with mental distress: female sex (AOR = 2.8, 95% CI: 1.50–3.16), rural residence (AOR = 1.96, 95% CI: 1.30–2.95), poor social support (AOR = 2.03, 95% CI: 1.20–3.42), not participating in regular physical exercise (AOR = 3.28, 95% CI: 1.68–6.37), never attending guidance and counseling services (AOR = 2.37, 95% CI: 1.12–4.99), and family history of mental illness (AOR = 2.65, 95% CI: 1.31–5.35) were found to be associated factors for mental distress with p-value < 0.05 in multivariable logistic analysis (**Table 5**).

**Table 5:** Bivariate and multivariate logistic regression analysis of factors associated with mental distress among private college students in Gondar town, Northwest Ethiopia, 2023 (n = 620).

Variables	Category	Mental distress		OR with 95% CI	
		Yes	No	COR	AOR
Sex	Male	134	168	1	1
	Female	193	125	1.94(1.40,2.67)	2.18(1.50,3.16) ***
Religion	Orthodox	275	246	1	1
	Muslim	30	33	0.81(0.48,1.37)	1.02(0.53,1.93)
	Protestant	16	7	2.04(0.83,5.05)	2.15(0.75,6.21)
	Others	6	7	0.77(0.25,2.31)	0.89(0.24,3.28)
Spiritual practice	Yes	299	276	1	1
	No	17	28	1.52(0.81,2.84)	1.29(0.68,2.86)
Residency	Urban	186	211	1	1
	Rural	141	82	1.95(1.39,2.73)	1.96(1.30,2.95) **
Living with	Both parent	130	128	1	1
	Single parent	73	50	1.44(0.93,2.22)	1.50(0.90,2.50)
	Lonely	82	64	1.26(0.84,1.90)	1.03(0.64,1.65)
	With spouse	22	29	0.75(0.41,1.37)	0.83(0.41,1.66)
	With friends	18	19	0.93(0.47,1.86)	0.62(0.29,1.35)
	Others	2	3	0.66(0.11,3.99)	0.52(0.08,3.46)
CGPA	Satisfactory	30	20	1.67(0.89,3.14)	1.38(0.66,2.90)
	Distinction	93	64	1.62(1.06,2.47)	1.69(1.05,2.71)
	Great distinction	109	103	1.18(0.80,1.74)	1.10(0.72,1.69)
	Very great distinction	95	106	1	1
Attending guidance and counseling	Yes	48	67	1	1
	No	37	20	2.58(1.34,4.99)	2.36(1.12,4.99) *
	Not available	242	206	1.64(1.08,2.48)	1.52(0.94,2.43)
Good prospect to find a job	Yes	247	237	1	1
	No	80	56	1.37(0.93,2.01)	1.34(0.86,2.10)
Alcohol ever use	Yes	210	164	1.41(1.02,1.95)	1.33(0.81,2.20)
	No	117	129	1	1
Alcohol current use	Yes	147	107	1.42(1.03,1.96)	1.51(0.92,2.47)
	No	180	186	1	1
Regular physical exercise	Yes	19	40	1	1
	No	308	253	2.56(1.45,4.54)	3.28(1.68,6.37) ***
Social support	Poor	143	58	1.97(1.85,4.76)	2.02(1.20,3.42) **
	Moderate	170	130	0.92(0.60,1.41)	0.78(0.49,1.24)
	Strong	54	65	1	1
Family history of mental illness	Yes	50	14	3.60(1.94,6.66)	2.64(1.31,5.35) **
	No	277	279	1	1

CGPA, cumulative grade point average; Hosmer and Lemshow goodness-of-fit test: p-value = 0.96.

*Significant at p-value 0.05, **Significant at p-value 0.01, ***Significant at p-value 0.001.

## Discussion

### Prevalence of mental distress

The overall prevalence of mental distress among private college nursing students was consistent with studies in Ethiopia (Samara) (53.2%) (16) and India (52%) (6). On the contrary, this study (52.7%) was higher than in previous studies conducted in Ethiopia (Gondar (40.9%) (19), Wollo (19.3%) (15), Hawassa (22.30%) (21), Mizan-Aman (29.2%) (18) and Jima (19.7%) (43)). This difference might be because only nursing students were included in this study, whereas previous studies included students from different fields. Profession-specific pressures on nursing students—such as fear of acquiring disease, feelings of inadequacy when confronting certain conditions, and sharing patients’ pain during clinical practicum—could increase the magnitude of mental distress (44, 45). In addition, it may also be that students in private colleges do not have the same protection as public university students and Gondar is also found near to war-affected areas. Furthermore, differences in assessment tools may explain the variation: many earlier studies used the SRQ-20, while this study used the Kessler-10, which considers both mild and moderate levels of distress as “distressed,” potentially inflating prevalence.

The prevalence in this study was also higher compared with studies in Somaliland (Hargeisa) (19.8%) (27), Egypt (17.1%) (24), Tanzania (13.9%) (46), Nigeria (29.6%) (8), India (34.8%) (31). This variation could be related to sex distribution: previous studies reported male predominance, while in the current study more than half of the participants were females (27). Sample size variation may also account for the differences.

However, the prevalence in the current study was lower when compared with studies in Jordan (65.7%) (47), France (75%) (48), Brazil (81.4%) (49) and China (90.86%) (10). This discrepancy may be explained by time variation, as most of those studies were conducted during the active expansion of the COVID-19 pandemic, when lockdowns may have increased mental distress (50, 51). In addition to this, instrumental variation (GHQ was applied to assess mental distress) and also, sample size variation would have contributed to this discrepancy, or it could be a real difference.

### Factors associated with mental distress

#### Gender

In this study, sex was significantly associated with mental distress. Female students were more than twice as likely to develop mental distress compared with male students (AOR = 2.18, 95% CI: 1.50–3.16). This finding is consistent with studies from Ethiopia (Debre-Birhan (52), Jimma (43), Hawassa (21), and Mizan-Aman (18), Egypt (24), Jordan (47), and China (10), which also showed higher levels of mental distress among female students. The possible reasons for these sex differences could be; biological variations, such as menstruation-related hormonal changes, as well as additional stressors such as household responsibilities and emotional pressures, which may contribute to higher rates of distress among female students compared with their male counterparts (18, 53, 54).

#### Residency

Regarding their residency, students who come from rural areas had nearly twice the odds of developing mental distress compared with town dwellers (AOR = 1.96, 95% CI: 1.30–2.95). This finding is supported by other studies (55, 56). Entering college requires adjustment at various levels, and rural students may need to cope with unfamiliar environments that are very different from their backgrounds. These challenges could provoke mental distress (57).

#### Counseling service and social support level

Students who did not attend guidance and counseling services were more than twice as likely to develop mental distress compared with those who did (AOR = 2.36, 95% CI: 1.12–4.99). This was consistent with findings from Tanzania (58) and Britain (59), which reported significant reductions in mental distress among students who used guidance and counseling services. This may be because counseling enables students to perceive reality more accurately and accept it.

In addition, this study also indicated that social support was a significant factor in mental distress. Students with poor social support were about twice as likely to experience mental distress compared with those with strong social support (AOR = 2.02, 95% CI: 1.20–3.42). This finding is consistent with studies from Ethiopia (19, 20), Tanzania (46), Ecuador (60), and Spain (61). Social support may promote health by buffering the adverse physiological effects of stress and providing a sense of belonging through communication (62).

#### Physical exercise

In this study, students who did not perform regular physical exercise were more than three times as likely to develop mental distress compared with those who exercised regularly (AOR = 3.28, 95% CI: 1.68–6.37). This close association may be explained by several biological mechanisms. Exercise may increase diversity in the gut microbiome, including genera within the phylum Firmicutes (63), and positively modulate the gut–brain axis through normal neurotransmitter production, including higher cerebral serotonin levels (64), and through neurogenesis (65, 66). Furthermore, exercise has been shown to enhance endorphin release, which decreases pain and may alleviate mental distress (63, 67, 68).

#### Family history of mental illness

Students who reported a family history of mental illness were more than twice as likely to experience mental distress compared with those who did not (AOR = 2.64, 95% CI: 1.31–5.35). Similar findings have been reported in other areas of Ethiopia—Gondar (19), Wollo (15), Axum (20), and Adama (69)—as well as in Egypt (24), showing that family history of mental illness is significantly associated with students’ mental distress. This may be explained by genetic factors, poor community perceptions of mental illness, treatment costs, and the limited availability of mental health services in the country (70).

## Limitations

This study has some limitations. Initially, the cross-sectional nature of the study design does not provide or confirm an actual cause-and-effect relationship. Furthermore, the data were collected by a self-administrative instrument and some of the variables (such as substance use and family history of mental illness) had required recall of history which might be prone to recall bias.

## Conclusion and recommendations

The overall prevalence of mental distress in this study was notably high, which means that more than half of the students were affected. Significant associated factors included female sex, rural residence, poor social support, lack of regular physical exercise, non-use of guidance and counseling services, and family history of mental illness. We recommend that the Ministry of Health and the Ministry of Education prioritize mental distress among students by developing and implementing remedial interventions. Colleges should enhance their guidance and counseling services by employing specialized counselors familiar with the stressors faced by healthcare students. Additionally, we recommend promoting regular physical exercise through on-campus fitness programs.

## Data Availability

The raw data supporting the conclusions of this article will be made available by the authors, without undue reservation.
