# Correction: Prevalence and associated factors of mental distress among private college nursing students in Gondar town, Northwest Ethiopia, 2023

**DOI:** 10.3389/fpsyt.2025.1752543

**Published:** 2025-12-09

**Authors:** Agegnehu Amare, Niguse Yigzaw, Bizuneh Tesfaye, Enguday Tirfeneh, Seblewongel Tinsae, Kiber Temesgen, Gebremeskel Mesafint

**Affiliations:** 1 Department of Psychiatry, Mizan-Tepi University, Mizan-Aman, Ethiopia; 2 Department of Psychiatry, College of Medicine and Health Sciences, University of Gondar, Gondar, Ethiopia

**Keywords:** mental distress, private college, nursing students, Gondar, Ethiopia

Authors “Niguse Yigzaw, Enguday Tirfeneh and Seblewongel Tinsae” were erroneously spelled as “Ngusie Ygzaw, Ennguday Trfeneh and Seblewengel Tinsae”.

The original version of this article has been updated.

